# Initial computed tomography imaging details during first-line systemic therapy is of significant prognostic value in patients with naïve, unresectable metastatic renal cell carcinoma

**DOI:** 10.1371/journal.pone.0177975

**Published:** 2017-05-31

**Authors:** Sung Han Kim, Weon Seo Park, Sun Ho Kim, Ho Kyung Seo, Jae Young Joung, Kang Hyun Lee, Jinsoo Chung

**Affiliations:** 1 Department of Urology, Center for Prostate Cancer, National Cancer Center, Goyang, Korea; 2 Department of Pathology, Research Institute and Hospital of National Cancer Center, Goyang, Korea; 3 Department of Radiology, Research Institute and Hospital of National Cancer Center, Goyang, Korea; Institute of Bioengineering and Nanotechnology, SINGAPORE

## Abstract

**Purpose:**

We aimed to determine the prognostic significance of computed tomography imaging parameters of unresectable primary renal tumor lesions, obtained at baseline and at first follow-up, on overall survival in naïve, unresectable metastatic renal cell carcinoma patients during first-line systemic therapy.

**Materials and methods:**

Clinicopathological parameters of 56 patients treated between 2007 and 2015, including imaging parameters (such as the longest tumor diameter, necrotic area diameter, and attenuation in primary renal tumor lesions on baseline vs. follow-up computed tomography), were retrospectively reviewed to derive predictive factors of overall survival. The best overall response was measured according to the RECIST v1.1.

**Results:**

The median treatment period was 206.3 days and the median follow-up was 14.6 months. Forty-four (78.6%) patients progressed after a median 4.6 months of progression-free survival, and 6 (10.7%) patients survived with a median overall survival of 12.5 months. Multivariate analysis showed that the baseline tumor diameter (hazard ratio [HR] 0.903) and mean attenuation (HR 0.936), change of tumor diameter (HR 0.714) and necrosis diameter (HR 0.861), change in the percentage of tumor diameter (HR 1.483) and of necrosis diameter (HR 1.028) between baseline and follow-up computed tomography images; treatment duration (HR 0.986) and baseline serum hemoglobin (HR 1.790) and albumin level (HR 0.060) were significant factors for overall survival (*p*<0.05).

**Conclusion:**

The study showed that baseline and first follow-up computed tomography findings of primary renal lesions during first-line systemic therapy are useful and significant predictors of OS in patients with naïve unresectable mRCC.

## Introduction

Over the past 2 decades, the advent of molecular targeting agents has greatly improved the prognoses of advanced renal cell carcinoma (RCC), producing higher therapeutic response rates as well as longer progression-free survival (PFS) and overall survival (OS) compared to those of previous immunotherapeutic strategies [1,2]. As a result, the standard treatment has shifted from immunotherapy (IT) to targeted therapy (TT), in which interferon (IFN)-α in combination with other TTs and interleukin (IL)-2 alone are utilized as first-line systemic therapies in selected metastatic RCC (mRCC) patients [3]. However, the prognosis of advanced RCC remains disappointing; stages III and IV RCC have 60% and 10% 5-year disease-specific survival rates, respectively, in spite of such treatments [4]. While improved OS trends mirror those of PFS, the survival benefit from TT is still limited, with a median of less than 2–3 years, and is often not statistically significant. Therefore, researchers have attempted to devise the best therapeutic protocols with a diverse combination of systemic therapies in order to improve OS.

In advanced RCC, patients with unresectable mRCC reportedly have worse OS rates than those that undergo cytoreductive nephrectomy combined with either IT or TT (IT and TT without cytoreductive nephrectomy: 3 and 13 months, respectively; compared to IT and TT with cytoreductive nephrectomy: 4 and 19 months, respectively) [5]. The unfavorable prognosis of unresectable mRCC patients has been attributed to the patients’ poor general conditions that make them unable to tolerate the total dose of first-line systemic therapeutic agents required, and by large tumor burdens that make surgery impossible. To better estimate the prognoses of naïve unresectable mRCC patients, several prognostic models for mRCC as well as known significant predictive factors for OS have been used by clinicians for the purposes of selecting patients more likely to benefit from ongoing therapy, promptly preparing additional therapeutic plans with more accurate first-line therapy evaluation tools, and saving time by initiating subsequent therapy earlier within the treatment window.

Among evaluation tools for tumor response, the benefit of contrast-enhanced computed tomography (CT) was demonstrated using recently created multiple response criteria [6–8] following routine use by mRCC patients during follow-up visits [9–11]. A significant correlation between tumor size or enhancing attenuation and clinical outcome was shown in mRCC patients with TT [6,10,12]. Follow-up CT after 1 or 2 cycles of initiation of systemic therapy had a closer correlation with prognosis in terms of therapeutic responsiveness than imaging studies performed during other follow-up periods [13–15]. The degrees of responsiveness of unresectable primary renal lesions, as well as several baseline patient parameters such as performance status and laboratory findings, were found to be important for the prediction of therapeutic outcomes [9,16–22].

In this study, patients with naïve mRCC and unresectable primary renal lesions without nephrectomy were enrolled; the Response Evaluation Criteria In Solid Tumors (RECIST) were used to determine the efficacy of systemic therapy [8,19]. The clinicopathological parameters during first-line systemic therapy, including imaging information on both baseline CT and first follow-up CT, were analyzed with the aim of identifying significant predictive factors for OS.

## Material and methods

### Ethical statements

All study protocols were conducted according to the ethical guidelines of the World Medical Association Declaration of Helsinki: Ethical Principles for Medical Research Involving Human Subjects. All the enrolled patients’ medical records were de-identified and analyzed anonymously. This study was approved by the Institutional Review Board of the Research Institute and Hospital National Cancer Center (IRB No. NCCNCS 13–816). The IRB waived the requirement for written informed consent.

### Patient selection

The mRCC patients with unresectable primary renal lesions without nephrectomy, treated between January 2007 and March 2015, were enrolled from the prospectively recorded RCC database of the hospital. Patients who had no follow-up CT during first-line systemic therapy, discontinued systemic therapy owing to adverse side effects, refused therapy, had a past history of invasive surgical or local treatment for RCC (including nephrectomy, embolization, and radiation therapy), had bilateral RCCs, and had incomplete information on past history of treatment for RCC were excluded. Ultimately, 56 patients with mRCC who had not undergone nephrectomy were enrolled and followed until July 2015.

### Treatment regimen and evaluating tools

The choice of systemic therapy (either IT or TT) was at the discretion of the treating urologist (J.C.) with consideration of each patient’s histopathology, disease status, performance status, coverage by the National Health Insurance System, and the wishes of the patient and his/her family after comprehensive discussion about the anticipated efficacy and adverse events of each agent.

For combination IT, triple or quadruple regimens were administered as follows [23]: IL-2, 20 MIU/m^2^ on days 3–5 of weeks 1 and 4, and 5 MIU/m^2^ IL-2 on days 1, 3, and 5 of weeks 2 and 3; IFN-α, 6 MIU/m2 on day 1 of weeks 1 and 4 and on days 1, 3, and 5 of weeks 2 and 3, and 9 MIU/m^2^ on days 1, 3, and 5 of weeks 5–8; fluorouracil-5 (5-FU), 750 mg/m^2^ once weekly during weeks 5–8; with (quadruple) or without (triple) vinblastine at 0.1 mg/kg once weekly during weeks 5–8. The dual combination regimen of vinblastine plus IFN-α was administered as IFN-α at 9 MIU/m^2^ on days 1, 3, and 5 each week, and vinblastine at 0.1 mg/kg every 3 weeks. For sunitinib, each cycle consisted of 50 mg daily oral intake for 4 weeks followed by a 2-week hiatus; for sorafenib, each cycle consisted of 400 mg twice daily oral intake for 6 weeks; and for pazopanib, each cycle consisted of 800 mg once daily oral intake for 6 weeks.

After every 2 cycles (or each cycle of quadruple or triple combination IT) of systemic therapy, patients underwent a total physical evaluation with blood tests and radiologic examinations, including CT and/or positron emission tomography-CT, as well as bone scans, to evaluate the treatment response according to the RECIST (version 1.1) [8]. Treatment was continued until disease progression was detected. Our CT imaging protocol was published previously [24]. CT imaging was performed using 4-channel and 16-channel multidetector CT scanners (Mx 8000: Marconi Medical Systems, Tel Aviv, Israel; and LightSpeed Pro 16: GE Healthcare, Milwaukee, WI, respectively). A CT scan was performed before contrast injection. Scanning for early phase images began 35 seconds after the start of intravenous contrast injection either from the lower thorax to the lower pelvis or from the neck to the upper abdomen. The remaining scanning and CT measurements were described previously [24].

### Statistical methods with imaging parameters

**Fig 1:**
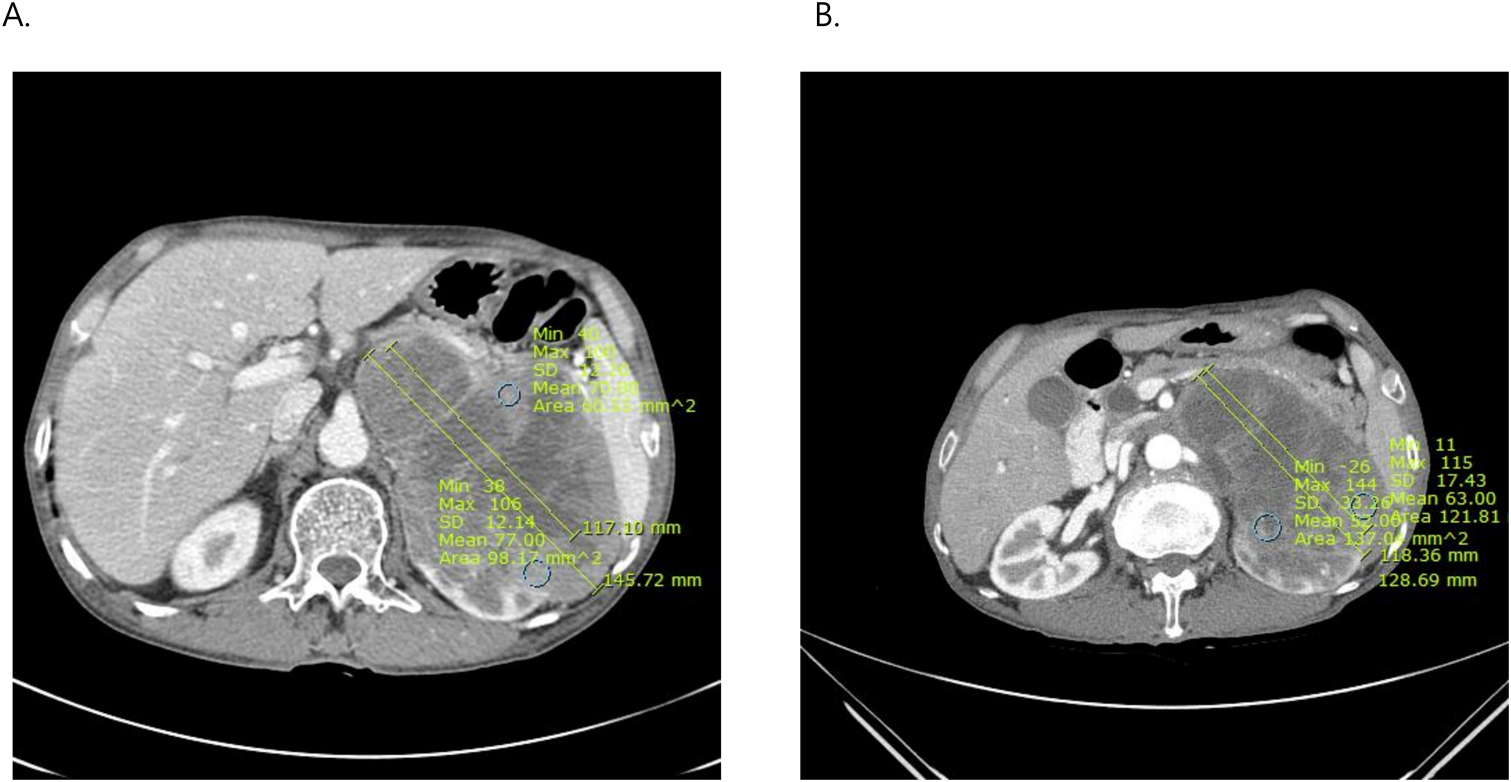
Representative changes of tumor size and necrosis size with attenuation on first follow-up contrast-enhanced computed tomography (CT) scans in metastatic renal cell carcinoma (RCC) patients with unresectable primary RCC. A. Axial CT image before sunitinib therapy showed a 14.5 cm-sized enhancing left renal mass with hilar invasion and presence of venous tumor thrombi. B. Compared to the CT image before targeted therapy, the CT image after 2-cycles of sunitinib showed that the tumor’s size had decreased (12.9 cm) with increased necrosis (11.8 cm) and decreased attenuation (from 77 Hounsfield units to 52 Hounsfield units).

In order to assess tumor response, all patients who underwent baseline CT within 4 weeks prior to starting therapy, and their first follow-up CT after the first cycle of IT (quadruple or triple combination) or first 2 cycles of TT (including dual combination IT) were considered available for review by a uroradiologist with 10 years of experience (SK). The imaging CTs were performed using the same protocol. For each patient, the largest horizontal diameter of the primary renal lesion with its mean attenuation, as well as the largest diameter of the necrotic area within the selected primary renal lesion, were measured on baseline CT and first follow-up CT. If multiple renal tumors existed within the primary renal lesion, the largest tumor with the longest diameter was chosen for evaluation, along with its necrotic area (these was referred to as ‘largest diameter of tumor’ and ‘largest diameter of necrosis’, respectively). Mean tumor attenuation was measured using an automated volume of interest analysis (*syngo* CT Oncology; Siemens Healthcare, Malvern, PA, USA), encompassing the entire tumor, by one independent reviewer (SHK) who was blinded to this study, and was expressed as Hounsfield units (HU) at contrasted renal phase for the baseline and first follow-up CTs (Fig 1) [24]. Tumor attenuation was determined by selecting and measuring the largest and most enhancing tumor on CT that had the least necrotic portion and no calcified areas. The percent tumor diameter change was referred to as the ‘primary renal lesion diameter percentage’ and calculated as the percentage change between the baseline and first follow-up CT; the change of necrosis diameter in primary renal lesion, referred to as the ‘primary renal lesion necrosis diameter change’, was calculated similarly. The equation used was [baseline CT − first follow-up CT] / baseline CT × 100%). The primary renal lesion necrosis diameter percentage change ranged from negative values to zero. All the imaging parameters (including primary renal lesion tumor diameter, necrosis diameter, and attenuation [HU] number) were repeatedly measured by one urologic oncologist (SHK) at 6-week intervals following the first reading.

Statistical analyses were performed using the Stata software (Release 9.2, StataCorp, College Station, TX, USA). The test-retest reliability method for calculating the intraclass correlation coefficient (ICC) was performed to rule out intra-operator variations [25]. Scatter diagrams and Bland-Altman plots were also constructed for intrapersonal variations. PFS and OS estimates were assessed using Kaplan–Meier analysis. Univariate and multivariate Cox regression models were employed to identify potential baseline prognostic variables for OS. Clinically important variables, such as the imaging parameters of baseline and first follow-up CTs, were subjected to multivariate analysis even if not found to be significant on univariate analyses. Additional cut-off values were determined based on statistically significant imaging parameters derived through multivariate analysis, as these were related to the prognosis of mRCC. A 2-sided *p*-value of <0.05 was considered significant on multivariate analysis.

## Results

**Fig 2:**
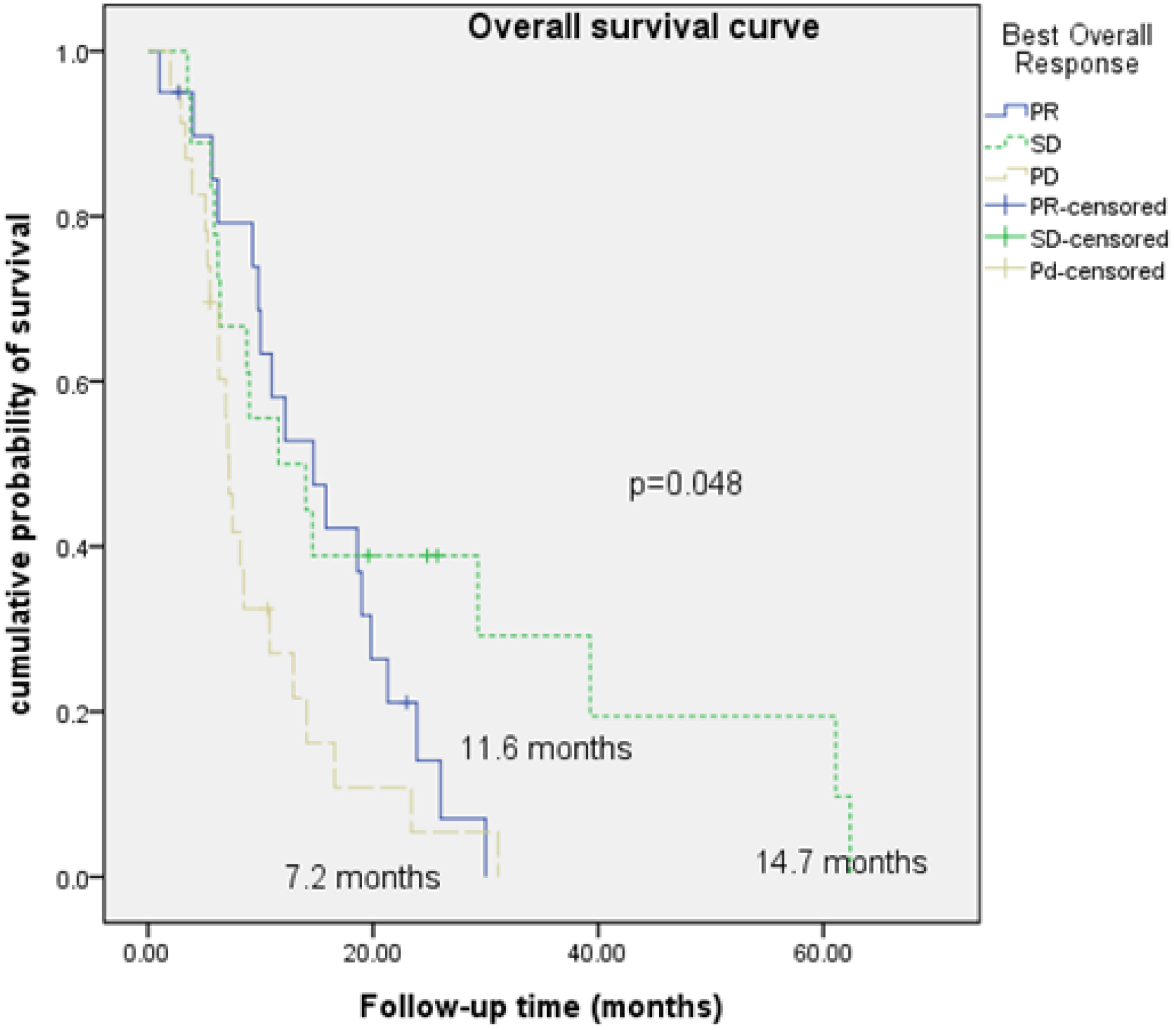
Overall survival curves among best overall response groups treated with first-line therapy.

During a median follow-up period of 14.6 months (range, 4–29 months) and a medical treatment period of 206.3 days (range, 110–954 days), 78.6% of patients (n = 44) experienced progressive disease (PD) during systemic therapy. Their disease control rates, objective response rates, and median PFS and OS rates were 57.1%, 23.2%, and 4.6 (4–31.8) and 12.5 (4–62.4) months, respectively (Table 1). The best overall responses according to the RECIST significantly differed after first-line systemic therapy, with median OS rates of 14.7, 11.6, and 7.2 months observed in the partial response (PR), stable disease (SD), and PD groups, respectively (*p*<0.05; Fig 2). Primary renal lesion tumor characteristics revealed a mean number of lesions of 2.0±1.0 (standard deviation), a primary renal lesion tumor median diameter of 9.3 (range, 1.7–15.8) cm, a primary renal lesion tumor median necrosis diameter of 4.9 (range, 0–12.4) cm, and a primary renal lesion tumor mean attenuation of 107.0 (range, 48–189) HU. Other characteristics are listed in Table 1.

**Table 1:** Patient baseline demographics (N = 56 patients with 62 primary lesions).

Parameters	Median, (range)
Age (yrs)	60.4 (26.1–80.8)
Sex, male/female, n (%)	46/10 (82.1/17.9)
Follow-up time (mos.)	14.6 (4–62.4)
Body mass index (cm^2^/m)	22.5 (13.9–30.3)
Karnofsky performance score	100 (90–100)
MSKCC, n(%): Favorable risk	16 (28.6)
Intermediate risk	38 (67.9)
Poor risk	2 (3.6)
Heng, n(%): Favorable risk	13 (23.2)
Intermediate risk	40 (71.4)
Poor risk	3 (5.4)
Histopathology, n (%): clear cell type	54 (96.4)
Non-clear cell type	2 (3.6)
Sarcomatoid component (n,%)	3 (5.4)
Laboratory findings	
Hemoglobin (g/dL)	11.9 (7.7–19.3)
Platelet (/uL)	296.9K (91K-614K)
Lactate dehydrogenase	247.1 (7–1456)
Calcium (mg/dL)	9.3 (3.4–14.5)
Albumin (g/dL)	3.8 (2.7–5.7)
Lymphocyte (%)	21.8 (0.2–56)
Neutrophil (/uL)	4936.3 (2289–11557)
Clinical T stage T1	4 (7.1)
T2	14 (25.0)
T3	15 (26.8)
T4	19 (33.9)
Tx	4 (7.1)
Clinical N stage N0	17 (30.4)
N1	22 (39.2)
Nx	17 (30.4)
Fuhrman nuclear grade, n (%): 1	5 (15.2)
2	16 (48.4)
3	6 (18.2)
4	6 (18.2)
Unknown	20
Metastatic lesions (mean ±SD)	2.0 ± 1.0
Metastatic organs (median, range)	2.0 (0–4)
Lung, n (%)	50 (89.3)
Liver, n (%)	13 (23.2)
Lymph nodes, n (%)	28 (50.0)
Bone, n (%)	22 (39.3)
Brain or other sites, n (%)	4 (7.1)
Treating agent, n (%): Immunotherapy	11 (19.6)
Sunitinib	33 (58.9)
Sorafenib	4 (7.1)
Pazopanib	8 (14.3)
Treatment duration of first line therapy (days.)	206.3 (60–954)
Subsequent therapy, n (%)	22 (39.3)
Second-line target therapy	22 (39.3)
Embolization	3 (5.4)
Radiation therapy for metastasis	12 (21.4)
Metastatectomy	9 (16.1)
Tumor characteristic of primary renal lesion in baseline CT imaging	
Number of lesions	1.2 (1–3)
PRL tumor diameter (median, range; cm)	9.3 (1.7–15.8)
Presence of necrosis, n (%)	51 (91.1)
PRL tumor necrosis diameter (median, range; cm)	4.9 (0–12.4)
PRL tumor mean attenuation (median, range; Hounsfield unit)	107.0 (48–189)
Presence of venous thrombi, n (%)	18 (37.5)
Best overall response after first-line therapy	
Partial response	13 (23.2)
Stable disease	19 (33.9)
Progressive disease	24 (42.7)
Secondary or further therapy, n (%)	19 (33.9)
Primary renal tumor necrosis diameter increase ≥10%	41 (73.2)
<10%	15 (26.8)
Progression, n (%)	44 (78.6)
Survival, n (%)	6 (10.7)

MSKCC: Memorial Sloan Kettering Cancer Center; PRL, primary renal lesion

The test-retest method showed that the primary renal lesion tumor diameter and necrosis diameter had ICCs of 0.852 and 0.794, respectively, whereas the primary renal lesion mean attenuation had an ICC 0.568 (S1 Table). The scatter plots of mean attenuation at baseline and first follow-up CTs, as well as the Bland-Altman plots of the primary renal lesion tumor diameter, tumor mean attenuation, and tumor necrosis diameter are shown in S1 and S2 Figs. The primary renal lesion mean tumor attenuation numbers showed wide intrapersonal variability between the first and second readings.

Univariate analysis revealed that baseline neutrophil level, albumin level, hemoglobin level, lymphocyte level, Treatment duration, MSKCC favorable risk, primary renal lesion tumor diameter, primary renal lesion tumor necrosis diameter, primary renal lesion tumor mean attenuation, primary renal lesion tumor necrosis diameter change, primary renal lesion tumor mean attenuation change, primary renal lesion tumor diameter change, primary renal lesion tumor necrosis diameter change percentage, primary renal lesion tumor mean attenuation change percentage, primary renal lesion tumor diameter change percentage were significant risk factors for OS (*p*<0.05, S2 Table). The multivariate analysis showed that primary renal lesion tumor diameter (hazard ratio [HR] 0.903, 95% confidence interval [CI] 0.847–0.963) and mean attenuation (HR 0.936, CI 0.905–0.967), change of primary renal lesion tumor diameter (HR 0.714, HR 0.561–0.908) and necrosis diameter (HR 0.861, CI 0.781–0.949), change in the percentage of primary renal lesion tumor diameter (HR 1.483, CI 1.146–1.919) and of necrosis diameter (HR 1.028, CI 1.009–1.047) between baseline and follow-up CT images; treatment duration (HR 0.986, CI 0.979–0.993) and baseline serum hemoglobin (HR 1.790, CI 1.149–2.790) and albumin level (HR 0.060, CI 0.010–0.341) were significant factors for OS (*p*<0.05, Table 2).

**Table 2:** Cox regression analysis of predictive factors of overall survival.

	Univariate analysis				Multivariate analysis			
	95.0% C.I				95.0% C.I			
	Hazard ratio	Lower limit	Upper limit	p-value	Hazard ratio	Lower limit	Upper limit	p-value
Baseline neutrophil level	1.000	1.000	1.000	0.001	1.000	0.999	1.000	0.233
Baseline albumin level	0.600	0.378	0.953	0.030	0.060	0.010	0.341	0.002
Baseline hemoglobin level	0.853	0.757	0.963	0.010	1.790	1.149	2.790	0.010
Baseline lymphocyte level	0.969	0.944	0.996	0.024	0.988	0.889	1.099	0.826
Treatment duration	0.995	0.993	0.997	0.001	0.986	0.979	0.993	0.001
MSKCC favorable risk	0.494	0.253	0.965	0.039	0.666	0.170	2.603	0.559
Baseline PRL tumor diameter	1.003	0.995	1.012	0.453	0.903	0.847	0.963	0.002
Baseline PRL tumor necrosis diameter	1.009	0.996	1.021	0.175	1.038	0.984	1.095	0.168
Baseline PRL tumor mean attenuation	0.997	0.988	1.005	0.430	0.936	0.905	0.967	0.001
PRL tumor necrosis diameter change	1.010	0.994	1.026	0.245	0.861	0.781	0.949	0.002
PRL tumor mean attenuation change	1.003	0.995	1.011	0.474	0.964	0.861	1.078	0.517
PRL tumor diameter change	1.018	1.001	1.035	0.037	0.714	0.561	0.908	0.006
PRL tumor necrosis diameter change percentage	1.002	0.998	1.005	0.348	1.028	1.009	1.047	0.003
PRL tumor mean attenuation change percentage	1.002	0.993	1.012	0.630	1.043	0.921	1.182	0.504
PRL tumor diameter change percentage	1.020	1.004	1.035	0.012	1.483	1.146	1.919	0.003

C.I., confidence interval; MSKCC, Memorial Sloan Kettering Cancer Center criteria; PRL, primary renal lesion

**Fig 3:**
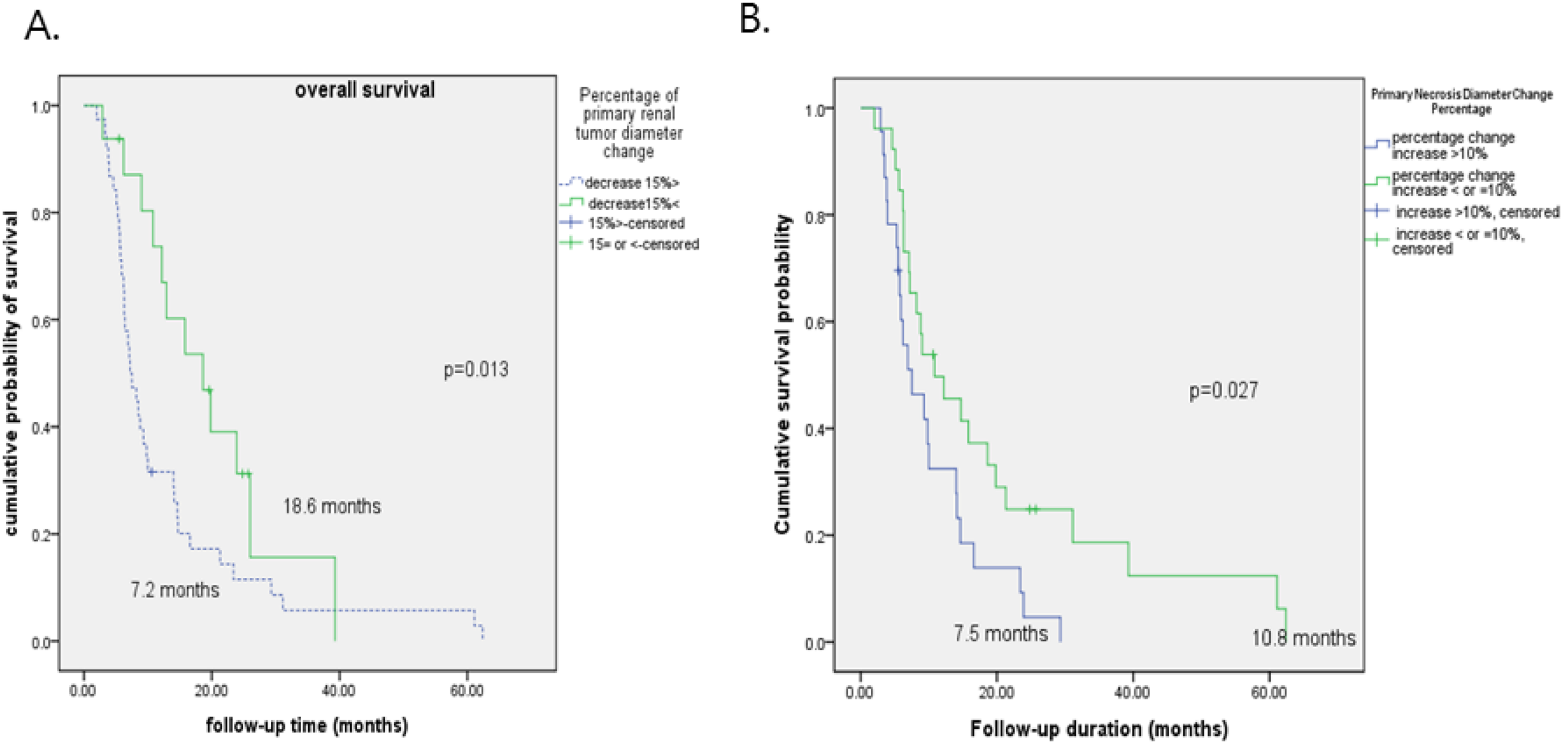
Overall survival curves comparing response groups according to the changes in the percentages of (A) the primary tumor diameter and (B) the primary tumor necrosis diameter from baseline computed tomography (CT) to first follow-up CT during first-line therapy.

Additionally, Kaplan-Meier analysis revealed a significant difference in OS between patients exhibiting a 15% decrease in the percentage of the primary renal lesion diameter (<15% [7.2 months] vs. ≥15% [18.6 months]), as well as a 10% change in the percentage of the necrotic area diameter (<10% [7.5 months] vs. ≥10% [10.8 months]) on baseline CT vs. first follow-up CT during first-line systemic therapy (N = 41, 73.2%) and those with a >10% change (N = 15, 26.8%) (*p* = 0.027, Fig 3). showed a significant difference in OS rates (*p*<0.05, Fig 3).

## Discussion

Considering an increase in the number of patients diagnosed with naïve unresectable mRCC, as well their dismal prognostic outcome [5], this study focused on the predictive parameters of OS during early first-line systemic therapy and on the clinical significance of primary renal lesion imaging parameters with respect to baseline and first follow-up CT. Using data from these 2 CT scans, clinicians can potentially identify patients who may respond to first-line systemic therapy (based on predicted OS) quickly and easily, and can also provide suitable alternatives to patients less likely to benefit.

Imaging parameters, particularly the changes observed between baseline CT and first follow-up CT during therapy, are clinically meaningful when compared to other timed contrast-enhanced CTs used to assess tumor responses based on RECIST, and are easy to obtain in outpatient clinics [13,15]. Moreover, the primary renal lesion was selected to test its significance for prognosis and its responsiveness to therapy. Its diameter and mean attenuation numbers on baseline CT, as well as changes in the diameters of the overall lesion and the necrotic area on first follow-up CT, provide significant prognostic information when treating naïve unresectable mRCCs during first-line therapy (*p*<0.05, Table 2). Previous studies showed that the primary renal lesion does not respond well with RECIST to treatment comparing to metastatic lesions. The authors of these studies recommended that metastatic lesions should be the focus of therapeutic strategies while suggesting that applying RECIST to primary renal lesions was not indicative of response rates and prognostic outcomes following systemic therapy [20,26,27]. However, evaluating primary renal lesion responses to systemic therapy, as we did in our study, is important because more than half of the patients with mRCC were ineligible for cytoreductive nephrectomy [28] and the primary renal lesion is the mainly tumor lesion in mRCC; hence, their primary renal tumor lesions were treated in situ with systemic therapy. Despite previous doubts about the ability of the RECIST to accurately determine tumor shrinkage and intratumoral changes owing to the unique complexities of specific tumors and their morphologic changes during systemic therapy [6,29,30], we found that the RECIST was suitable for evaluating the effects of both IT and TT on intratumoral changes in primary renal lesion, especially on necrosis in primary renal lesions.

First CT images showed treatment response and disease burden states during early therapy that were then compared to baseline states. Such modalities may complement other known prognostic models such as the MSKCC and Heng risk models, whose parameters are based on baseline clinicopathological parameters of nephrectomized mRCCs without considering the primary renal lesion or the patients’ general condition [12,13,31,32]. Clinicians can estimate the prognosis of mRCC patients by examining tumor responses sooner and with greater accuracy during treatment in order to plan appropriately [6,9].

We focused on the primary renal lesions because of their responsiveness compared to other tumor sites, including metastases, as they tend to be much larger than the other lesions and are easy to assess for purposes of determining the response to systemic therapy [12,20]. While the mean attenuation number representing the degree of enhancement and tumor density of the baseline primary renal lesion was a significant independent predictor of OS on multivariate analysis, the mean change in attenuation during therapy was not consistent with that found by Smith et al. [33]. We attribute this to the small sample size and intra-operator variability in measuring the enhancement within the primary renal lesions (S1 Table, S1 and S2 Figs) [25]. However, greater primary renal lesion volume shrinkage was previously shown to be correlated with increased necrosis and loss of enhancement [12,30]. Further large-scale prospective studies are required to ascertain the clinical importance of the attenuation numbers and their shifts during follow-up CTs with respect to the prognoses of patients with mRCC.

The changes in primary renal lesion tumor characteristics on the first CT during first-line systemic therapy were significantly related to both the objective response rate (CR + PR) and the clinical benefit (CR + PR + SD) according to the RECIST; the best measure of OS may be reflected by the greatest decrease in primary renal lesion tumor burden [18]. Additionally, analyses of histology and tumor diameter changes showed a significant correlation between TT and improvement in clear cell mRCC in this study (Pearson coefficient = 0.439; Kendall’s tau B = 0.244; *p*<0.05), whereas the nuclear grade and other imaging parameters such as tumor necrosis were not significant on correlation analyses (*p*>0.05; data not shown).

As for the morphological characteristics of primary renal lesion and their changes during therapy, the best therapeutic efficacy among naïve unresectable mRCC patients can be expected when the baseline size of a primary renal lesion tumor is smaller (hazard ratio [HR] = 0.903, *p* = 0.002) with a smaller necrotic portion (HR = 1.038, *p* = 0.168) and a higher attenuation number (HR = 0.936, *p* = 0.002); these parameters signify a small tumor cell density with high vascularity, making systemic therapy (especially with vascular endothelial growth factor-targeting agents) ideal. When the changes of tumor diameter (HR 0.714) and necrosis diameter (HR 0.861) in the primary renal lesion tumor is greater during first systemic therapy, better survival prognosis is observed (*p*<0.05), whereas greater changes in tumor diameters (HR 1.483) and necrosis diameter percentage changes (HR 1.028) predict poor therapeutic outcomes. Gradually increasing necrotic primary renal lesion portions indicate a poorer response to systemic therapy, resulting in worse clinical outcome [34]. This indicates that a decreasing proportion of active tumor cells are responsive to therapy, signifying therapeutic resistance. Lee et al. showed that macroscopic necrosis at baseline indicates a greater likelihood of a larger tumor, fast-growing metastatic disease, higher local stage, and higher tumor grade; this resulted in significantly poor disease- and progression-free survival rates [35]. Another study from Klatte et al. showed that the extent of necrosis (with a 20% cut-off in their case), rather than the actual presence of necrosis, influenced cancer-specific survival [36]. In our study, the arbitrary cut-off of a 15% decrease in the percentage of the primary renal lesion diameter (<15% [7.2 months] vs. ≥15% [18.6 months]), as well as a 10% change in the percentage of the necrotic area diameter (<10% [7.5 months] vs. ≥10% [10.8 months]) showed a significant difference in OS rates (*p*<0.05, Fig 3).

Tumor necrosis is a poor prognostic factor and portends a poorer response to systemic therapy in RCC patients [37–39]. The poor therapeutic response of tumors with extensive necrotic portions is a result of a more hypoxic state and less vascularity, which allows the tumor to escape the cytotoxic effects of systemic therapy through autophagy. Such tumors have a highly aggressive potential [37,38,40]. However, TT as first-line therapy for mRCC has distinct cytostatic and anti-angiogenic mechanisms compared to cytokine therapies; hence, mTOR inhibitors (e.g., temsirolimus) might be suitable for patients with extensive necrotic mRCC. Temsirolimus is indicated for poor risk mRCC with overactive mTOR pathways that are related to angiogenesis, cell proliferation, and metabolism. However, comparative studies of therapeutic responsiveness to mTOR inhibitor therapies as well as the extent of mTOR pathway activity in necrotic mRCC are required to evaluate treatment with other systemic therapies and targeting agents [41].

OS was also significantly affected by baseline serum albumin and hemoglobin levels. Albumin has already been shown to have a prognostic significance in many previous studies; these parameters reflect the patients’ general performance and nutritional statuses as well as their immune status, including systemic inflammation [42,43]. Elevated hemoglobin is also known to be a poor prognostic factor in relation to paraneoplastic erythropoiesis, with altered production of various hormones produced by the kidney including erythropoietin in clear cell mRCC [44].

Some limitations existed in this study, including the small population size, retrospective nature of the analysis (although it was based on a prospectively recorded RCC database), short-term follow-up duration, intra-observer variability in attenuation numbers obtained by CT measurements [25], possible technical inconsistencies in CT modalities, and the heterogeneity of our IT and TT patients. Our previous study showed that the Heng risk model had a better discriminating potential than the MSKCC model; this study did not evaluate the discriminating power because of the small number of patients [45]. However, we revealed a significant prognostic role for the morphological characteristics of the primary renal lesion on CT images during first-line therapy, as well as for systemic therapeutic agents. Our data are useful for clinicians in outpatient clinics, enabling them to better predict the prognoses of naïve, unresectable mRCC patients. None of the other well-known clinical factors such as T stage, age, sex, metastatic organs, histopathology, and paraneoplastic parameters had a significant effect on OS.

## Conclusion

We showed that baseline and first follow-up CT findings of primary renal lesions during first-line systemic therapy are useful and significant predictors of OS in patients with naïve unresectable mRCC. In conjunction with systemic therapies, baseline albumin level, primary renal lesion necrosis diameter, and primary renal lesion tumor diameter and necrosis change during first-line systemic therapy predict disease prognoses in a simpler and more efficient manner. Further studies with a prospective, multicenter, randomized design will be required for successful validation of our results.

## Supporting information

S1 Fig (PDF)

S2 Fig (PDF)

S1 Table (PDF)

S2 Table (PDF)

## Abbreviations

CI: confidence interval
FU: fluorouracil
HR: hazard ratio
HU: Hounsfield units
IFN: interferon
IL: interleukin
IT: immunotherapy
mRCC: metastatic renal cell carcinoma
MSKCC: Memorial Sloan Kettering Cancer Center
OS: overall survival
PD: progressive disease
PFS: progression-free survival
PR: partial response
RCC: renal cell carcinoma
SD: stable disease
TT: targeted therapy
