# Supplementary figures and images for: Initial computed tomography imaging details during first-line systemic therapy is of significant prognostic value in patients with naïve, unresectable metastatic renal cell carcinoma
Source: PLoS One. 2017 May 31;12(5):e0177975. doi: 10.1371/journal.pone.0177975 (PMC5451027; doi:10.1371/journal.pone.0177975)

Supplementary Figure 1. Scatter plot of mean attenuation.

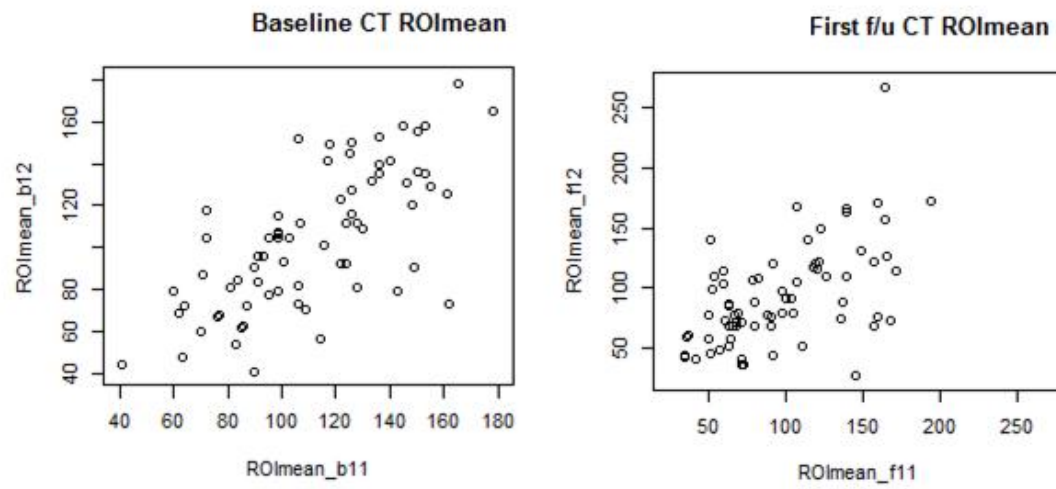

Supplement: S1 Fig — (PDF) [file pone.0177975.s001.pdf]
