# Supplementary material for: Initial computed tomography imaging details during first-line systemic therapy is of significant prognostic value in patients with naïve, unresectable metastatic renal cell carcinoma
Source: PLoS One. 2017 May 31;12(5):e0177975. doi: 10.1371/journal.pone.0177975 (PMC5451027; doi:10.1371/journal.pone.0177975)
Supplement: S1 Table — (PDF) [file pone.0177975.s003.pdf]

Supplementary Table 1. Results of intra-observer test-retest correlation tests

	Baseline CT				First follow-up CT			
	**ICC**	**lower**	**upper**	**p-value**	**ICC**	**lower**	**upper**	**p-value**
**PRL tumor diameter**	0.914	0.866	0.946	0.00E+00	0.852	0.772	0.906	0.00E+00
**PRL tumor mean attenuation**	0.697	0.553	0.800	7.66E-12	0.568	0.385	0.707	1.22E-07
**PRL tumor necrosis diameter**	0.648	0.482	0.770	1.96E-09	0.794	0.683	0.869	8.88E-16

CT: computed tomography, HU: Hounsfield units; ICC: intraclass correlation coefficient; PRL: primary renal lesion
