# Supplementary material for: Initial computed tomography imaging details during first-line systemic therapy is of significant prognostic value in patients with naïve, unresectable metastatic renal cell carcinoma
Source: PLoS One. 2017 May 31;12(5):e0177975. doi: 10.1371/journal.pone.0177975 (PMC5451027; doi:10.1371/journal.pone.0177975)
Supplement: S2 Table — (PDF) [file pone.0177975.s004.pdf]

Supplementary table 2. Univariate Cox regression analysis of predictive factors of overall survival

	Hazard ratio	95.0% Confidence Interval		p-value
		Lower limit	Upper limit	
Age	0.988	0.965	1.012	0.329
Body mass index	1.133	0.929	1.383	0.217
Male sex	1.367	0.655	2.851	0.405
Hypertension	1.869	0.728	4.803	0.194
Diabetes	1.120	0.269	4.670	0.876
Clear cell histology	0.260	0.059	1.134	0.073
Sarcomatoid component	0.308	0.092	1.030	0.056
Baseline laboratory findings				
Neutrophil	1.000	1.000	1.000	0.001
Hemoglobin	0.853	0.757	0.963	0.010
Platelet	1.002	1.000	1.005	0.080
Lactate dehydrogenase	1.001	0.999	1.002	0.472
Calcium	1.031	0.803	1.324	0.811
Albumin	0.600	0.378	0.953	0.030
Lymphocyte	0.969	0.944	0.996	0.024
Treatment duration	0.995	0.993	0.997	0.001
First line therapy immunotherapy				0.562
Sorafenib	1.554	0.530	4.558	0.422
Sunitinib	1.049	0.403	2.727	0.923
Pazopanib	1.844	0.486	7.001	0.369
Fuhrman nuclear grade 1				0.144
2	0.423	0.153	1.170	0.097
3	0.643	0.301	1.373	0.254
4	0.238	0.053	1.070	0.061
5	1.270	0.520	3.101	0.600
Clinical T1 stage				0.382
T2	0.236	.051	1.092	0.065

T3	0.406	.129	1.283	0.125
T4	0.557	.179	1.740	0.314
Tx	0.448	.143	1.406	0.169
MSKCC Poor risk				0.119
Intermediate risk	0.719	0.164	3.157	0.662
Favorable risk	0.494	0.253	0.965	0.039
Heng Poor risk				0.510
Intermediate risk	1.185	0.334	4.204	0.793
Favorable risk	0.717	0.366	1.406	0.333
No of baseline metastatic lesions	1.145	0.447	2.933	0.777
Baseline PRL tumor diameter	1.003	0.995	1.012	0.453
Baseline PRL tumor necrosis diameter	1.009	0.996	1.021	0.175
Baseline PRL tumor mean attenuation	0.997	0.988	1.005	0.430
PRL* tumor necrosis diameter change	1.010	0.994	1.026	0.245
PRL* tumor mean attenuation change	1.003	0.995	1.011	0.474
PRL* tumor diameter change	1.018	1.001	1.035	0.037
PRL* tumor diameter change percentage	1.020	1.004	1.035	0.012
PRL* tumor necrosis diameter change percentage	1.002	0.998	1.005	0.348
PRL* tumor mean attenuation change percentage	1.002	0.993	1.012	0.630
Lung metastasis	0.879	0.369	2.090	0.770
Liver metastasis	1.572	0.809	3.056	0.182
Bone metastasis	0.650	0.366	1.154	0.141
Lymph node	.827	0.468	1.460	0.512
Other	0.726	0.258	2.045	0.545

HU: Hounsfield unit; PRL: Primary renal lesion
